# Tailored modulation of the inflammatory balance in COVID-19 patients admitted to the ICU?—a viewpoint

**DOI:** 10.1186/s13054-021-03607-4

**Published:** 2021-05-25

**Authors:** Marnix Kuindersma, Rocio Ramos Diaz, Peter E. Spronk

**Affiliations:** 1 Department of Intensive Care Medicine, Gelre Hospitals, Albert Schweiterlaan 31, Apeldoorn, The Netherlands; 2 Department of Medical Microbiology, Gelre Hospitals, Albert Schweiterlaan 31, Apeldoorn, The Netherlands; 3 Expertise Center for Intensive Care Rehabilitation Apeldoorn (ExpIRA), Apeldoorn, The Netherlands

**Keywords:** COVID-19, Coronavirus, Dexamethasone, Inflammation

## Abstract

A growing consensus seems to be emerging that dexamethasone is a crucial component in the treatment of COVID-19-associated oxygen-dependent respiratory failure. Although dexamethasone has an undeniably beneficial effect on the inflammatory response in a subgroup of patients, the potential negative effects of corticosteroids must also be considered. In view of these negative effects, we argue that a one-size-fits-all dexamethasone approach may be potentially harmful in specific subsets of patients with COVID-19-associated ARDS. We propose a different individually tailored treatment strategy based on the patient’s inflammatory response.

## Background

The first COVID-19 wave of patients hit the Netherlands in March–April 2020. The ICU caregivers were initially overwhelmed by this new disease with signs and symptoms not experienced before. The autopsy studies soon revealed a heterogeneous disease with diffuse alveolar damage (DAD) acute fibrinous injury and organizing pneumonia in combination with endothelial cells activation causing microvascular thrombosis, pulmonary infarcts and venous thromboembolism [1, 2]. These findings are consistent with the diverse clinical presentation of severe COVID-19 marked by severe ARDS, activation of coagulation and clot formation, and in a subset of patients signs of a proinflammatory status [3].

To elucidate the pathophysiological mechanisms underlying this heterogeneous disease, much attention was initially paid to the inflammatory response in COVID-19 [4–6], which led to various therapeutic strategies that target this inflammatory response.

### Targeting inflammation in COVID-19

The circulating cytokine levels in COVID-19-associated ARDS are lower in comparison with ARDS of different origins [5, 6]. The clinical picture is not characterized by a systemic cytokine storm, and signs of hyperinflammation are only seen in a subset of patients [7]. These combined results suggest that COVID-19-associated ARDS could be a mixture of both hypo- and hyperinflammatory subtypes, as previously described in ARDS of different origins [8]. Nevertheless, the focus in research and treatment modalities has mainly been on hyperinflammation. Building on the assumption that the hyperinflammatory response is an important driver in the pathogenesis of activation of coagulation, as well as alveolar damage and fibrosis, several investigators advocated the use of inflammatory modulators, i.e., high-dose corticosteroids, immunoglobulins, anakinra or tocilizumab [9–11]. Corticosteroids are still the main and most frequently used intervention to modulate inflammation in ICU patients. Corticosteroids have been accepted with some restraint in ARDS patients, because of incongruent results regarding mortality reduction in the corticosteroid groups [12–15]. For example, the study by Meduri et al. showed significant ICU mortality reduction (20.6% vs 42.9%; p = 0.03) if corticosteroids were initiated early in the disease course with 1 mg/kg/day up to 28 days [15]. On the other hand, Steinberg et al. showed no mortality reduction in ARDS when treated with corticosteroids initiated after 7 days of mechanical ventilation [13]. In fact, a reduction in mortality due to corticosteroids as salvage therapy when initiated later in the disease course has not been demonstrated to date and could possibly even lead to higher mortality. Nevertheless, a meta-analysis of the largest studies demonstrated a positive effect of corticosteroids, if timely initiated, on mortality in ARDS (20 vs. 33%; p = 0.006) [16]. This was corroborated recently in a randomized trial that showed a significant mortality reduction of 15.3% (-25.9 to -4.9, p = 0.0047) in patients with ARDS if dexamethasone was initiated early with a dose of 20 mg dexamethasone IV daily for five days, followed by 10 mg daily for the next five days [17].

Although several issues are far from settled, i.e., optimal timing, dose and tapering schedules of corticosteroids in ARDS, early application of corticosteroids is currently recommended for ARDS as part of ESICM and SCCM guidelines [18]. We refer the reader to other sources for a more in-depth discussion of this subject in ARDS [19–21].

We would like to focus on the use of corticosteroids in COVID-19 now. The use of corticosteroids was discouraged in the first wave of COVID-19 for fear of prolonged viral shedding [22, 23]. The first study to show a positive effect of corticosteroids in COVID-19 was the pragmatic and randomized RECOVERY trial. This trial showed a reduced 28-day mortality in patients who received 6 mg dexamethasone on top of usual care in patients who were treated with supplemental oxygen (23.3% vs 26.2%) or were mechanically ventilated (29.3% vs 41.4%) [11]. Noteworthy was that in patients with short-term complaints (≤ 7 days) women and the elderly (> 70 years) there was only a trend toward a positive effect. This was most likely due to a lack of lack of statistical power, not a lack of efficacy. Unfortunately, the results of the RECOVERY trial halted other randomized studies on the effect of corticosteroids in COVID-19.

One of the terminated studies was the French CAPE COVID study. This randomized clinical trial was halted after enrollment of 149 patients with severe COVID-19 on the ICU (76 corticosteroids, 73 placebo). The endpoint of that study was treatment failure on day 21, i.e., death or persistent mechanical ventilation. Subjects were randomized between placebo or 200 mg/d hydrocortisone until day 7 and then decreased to 100 mg/d for 4 days and 50 mg/d for 3 days, for a total of 14 days. The preliminary results showed no significant difference between the placebo group (50.7%) and the corticosteroid group (42.1%) [24].

The Brazilian CoDEX study, a multicenter randomized study, was ceased after publication of the RECOVERY trial as well. All subjects in this trial were mechanically ventilated and randomized between 20 mg dexamethasone for 5 days (optionally followed by 10 mg for an additional 5 days or until discharge from ICU) and standard of care. Until halting the study, 299 patients had been enrolled and 148 were randomized to standard care and 151 to corticosteroids. The mean number of days free from mechanical ventilation, the primary study endpoint, was higher in the corticosteroid group than in the standard of care group (difference, 2.26; 95% CI 0.2–4.38; P = 0.04) [25]. A third study that was discontinued after the results of the RECOVERY trial was the corticosteroid study arm of the adaptive platform trial REMAP-CAP. In this study, patients were 1:1:1 randomized between a fixed dose of 50 mg intravenous hydrocortisone every 6 h for 7 days, 50 mg every 6 h for up to 28 days while in shock, or no corticosteroids. The trial was stopped after inclusion of 403 patients. On the primary endpoint of organ support-free days, treatment with a 7-day fixed-dose course of hydrocortisone or shock-dependent dosing of hydrocortisone, compared with no hydrocortisone, resulted in 93% and 80% probabilities of superiority, respectively, with regard to the odds of improvement in organ support-free days within 21 days [26]. A meta-analysis of the effect of corticosteroids on mechanically ventilated COVID-19 patients soon followed. In the 1703 patients analyzed, the summary OR for mortality was 0.70 (95% CI 0.48–1.01; P = 0.053) with OR of 0.64 (95% CI 0.50–0.82; P < 0.001) for dexamethasone. Subsequently, as of May 2020, routine use of 6 mg dexamethasone for 10 days in oxygen-dependent COVID-19 patients became the new standard of care [27]. Although this meta-analysis showed a convincingly positive effect of corticosteroids on 28-day all-cause mortality, the results of this meta-analysis should be interpreted with some caution due to the absence of stratification and incomplete information about some factors associated with outcome in the included trials [28].

In addition to the use of corticosteroid therapy, more recent studies of anti-IL-6 therapy have also shown reduced mortality in COVID-19-associated ARDS in general [29, 30]. The results of the aforementioned RECOVERY Group of anti-IL6 therapy in conjunction with corticosteroids showed a mortality reduction from 33 to 29% [30]. In the recently published REMAP-CAP trial, a 90-day survival gain (OR 1.61, 95% credible interval 1.25 to 2.08) was seen from anti-IL-6 therapy (i.e., tocilizumab/sarilimumab) in conjunction with corticosteroids for the entire group of severe COVID-19 patients as well [29]. What the exact role of anti-IL-6 therapy will prove to be, in light of the negative COVACTA trial, is to be further elucidated [31]. We may have to conclude that anti-IL-6 therapy only affects final outcome if it is combined with corticosteroids [32, 33].

Despite the potential benefits of corticosteroid modulation of the immune response in COVID-19-associated ARDS, its use also may carry significant risks including an increased risk of secondary infections and important long-term disadvantages such as muscle wasting [25, 34–37]. Although the benefits of dexamethasone for oxygen-dependent COVID-19 patients in general are well demonstrated, we do argue that the one-size-fits-all corticosteroid approach may be potentially harmful in specific subsets of COVID-19 patients. We propose a different individually tailored treatment strategy.

### Tailored immune modulation in COVID-19?

To identify the patients who may benefit most from inhibiting inflammation using dexamethasone, it is important to realize that COVID-19, similar to classical ARDS, is most likely a heterogeneous and complex disease, characterized by both pro-inflammatory and anti-inflammatory subphenotypes and highly variable histopathological subtypes ranging from diffuse alveolar damage (DAD), bronchopneumonia, necrotizing bronchiolitis to viral pneumonia [1, 8, 38].

**Fig. 1:**
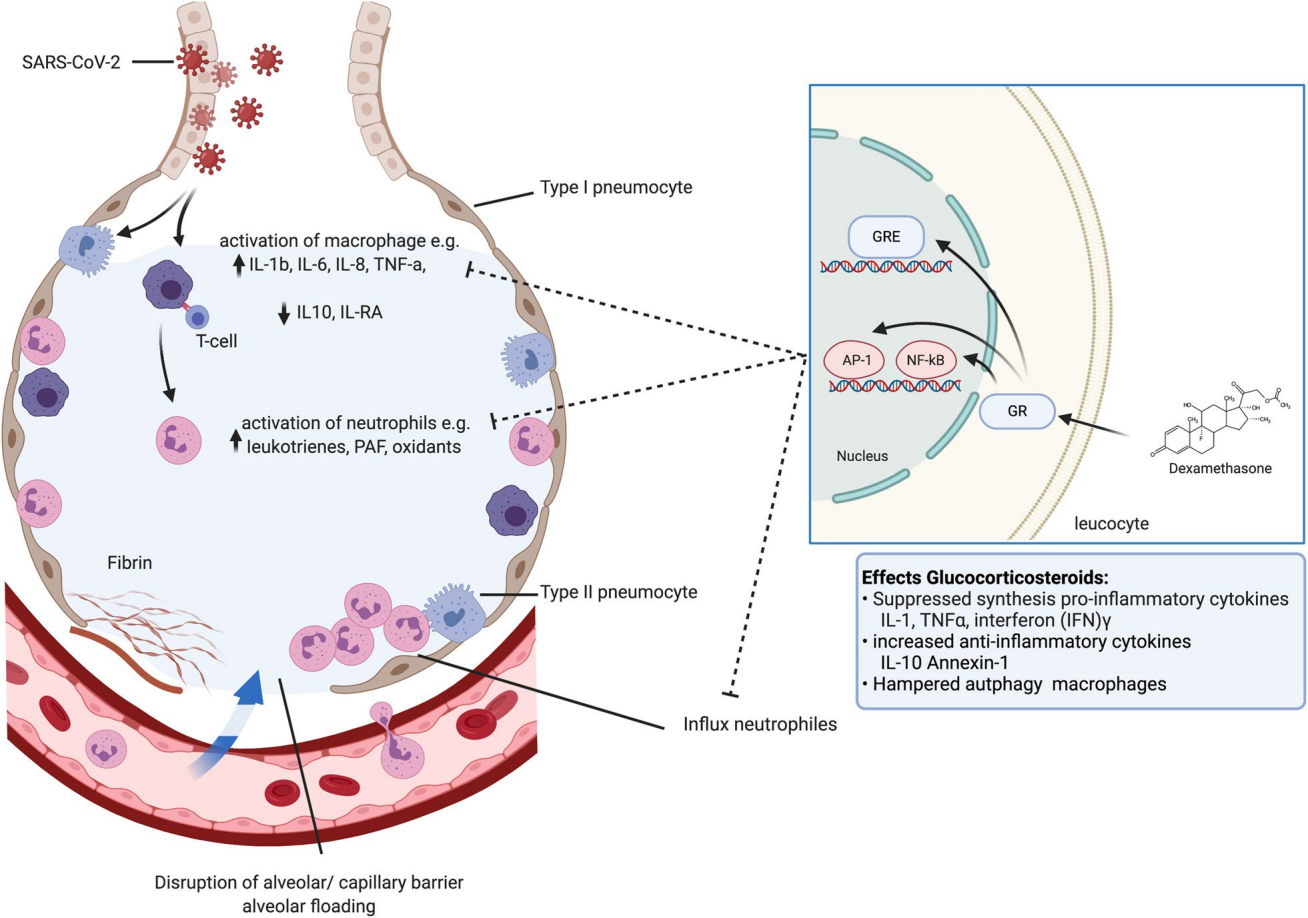
Inflammatory response and effects of corticosteroids on inflammation. This simplified cartoon illustrates the presumed pathophysiological steps during SARS-COV2 infection. The first step is the infection of type-2 pneumocytes and macrophages with SARS-CoV2 via the ACE-2 receptor, leading to the activation of alveolar macrophages. Activated macrophages release proinflammatory mediators (e.g., IL-1B, IL-6, IL-8 and tumor necrosis factor (TNF)-α) and suppress the anti-inflammatory mediators (e.g., IL-10, IL-1 receptor antagonist (RA)), promoting the infiltration of neutrophils in the alveolus. Toxic mediators are released by these neutrophils and cause further damage to the endothelial–epithelial barrier, initiating additional damage to the endothelial–epithelial barrier. Eventually, damage to these endothelial–epithelial cells, together with the loss of tight junctions and surfactant, leads to the influx of protein-rich fluid edema in the alveolar compartment which impairs gas exchange. Later, fibroblasts start to proliferate and growth factors are produced. This pro-inflammatory response can be suppressed by corticosteroids. Corticosteroids can freely diffuse the plasma membrane to bind to specific receptors. Upon binding to the glucocorticoid receptor (GR), the complex translocates to the nucleus where it interacts with glucocorticoid response elements (GRE), nuclear factor kB (NFKB) and activator protein-1 (AP-1). This interaction results in suppression of pro-inflammatory cytokines synthesis, influx and activation of neutrophils and impairment of macrophage autophagy. As a consequence, patients are more susceptible for secondary infections [3, 49, 50].

**Fig. 2:**
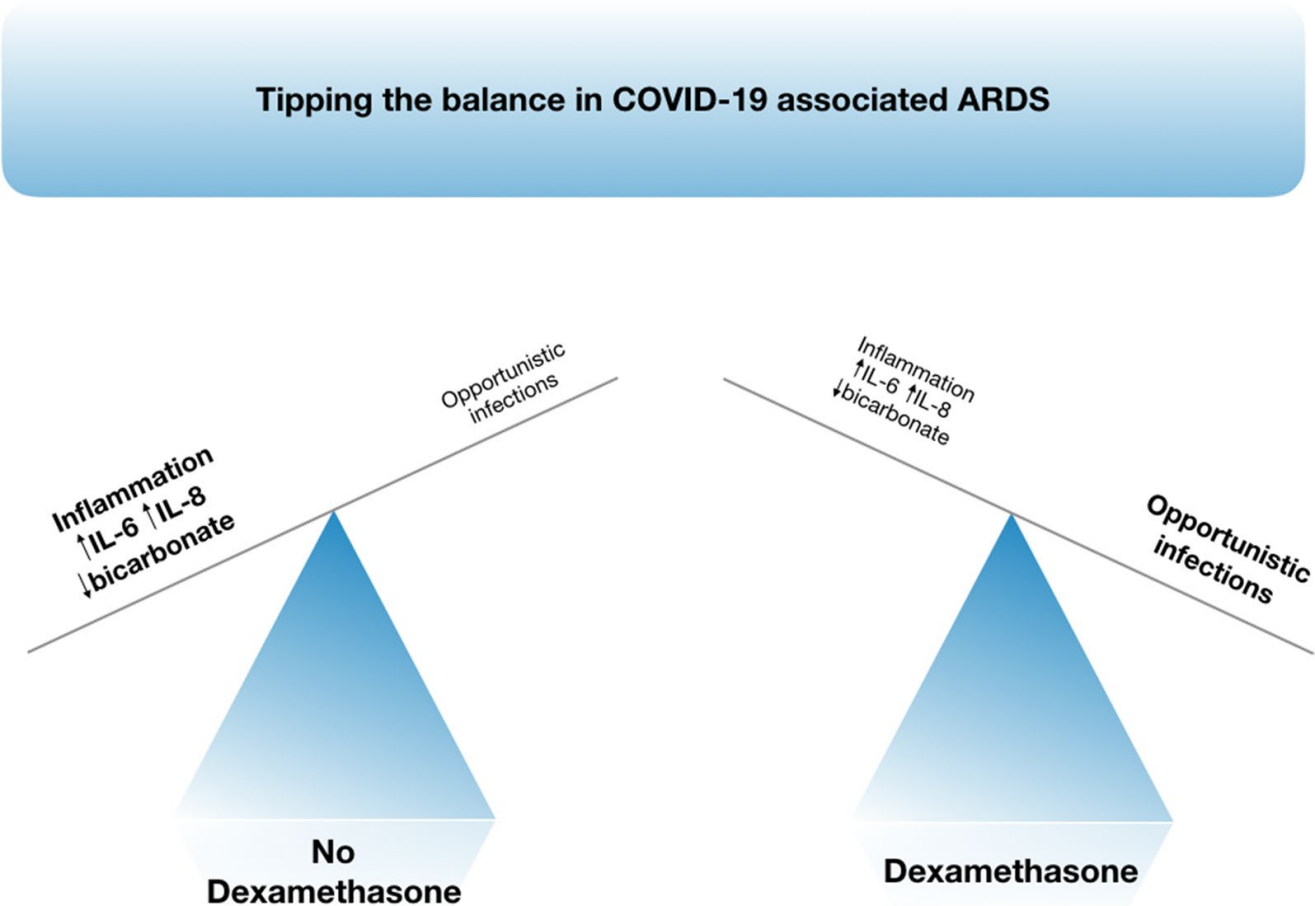
The balance between suppressing the inflammatory response in COVID-19-associated ARDS and the risk of secondary infections due to corticosteroid use

**Fig. 3:**
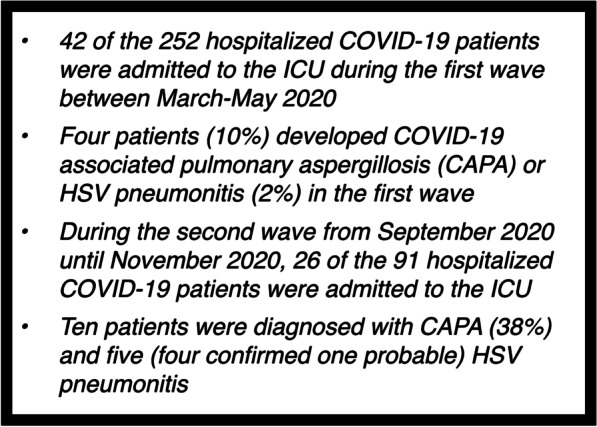
Box showing illustrative data of increased occurrence of secondary infections in our institution. All patients with a CAPA had a pulmonary infiltrate on conventional X-ray or chest CT, clinical deterioration and a positive VIRCLIA^®^ Aspergillus chemiluminescent immunoassay on bronchoalveolar lavage fluid, in accordance with the CAPA definition. HSV pneumonitis was diagnosed if there were a positive HSV PCR in BALF, a positive PCR in plasma and a cytological HSV staining in BALF in combination with respiratory deterioration with pulmonary infiltrate on conventional X-ray or chest CT

**Fig. 4:**
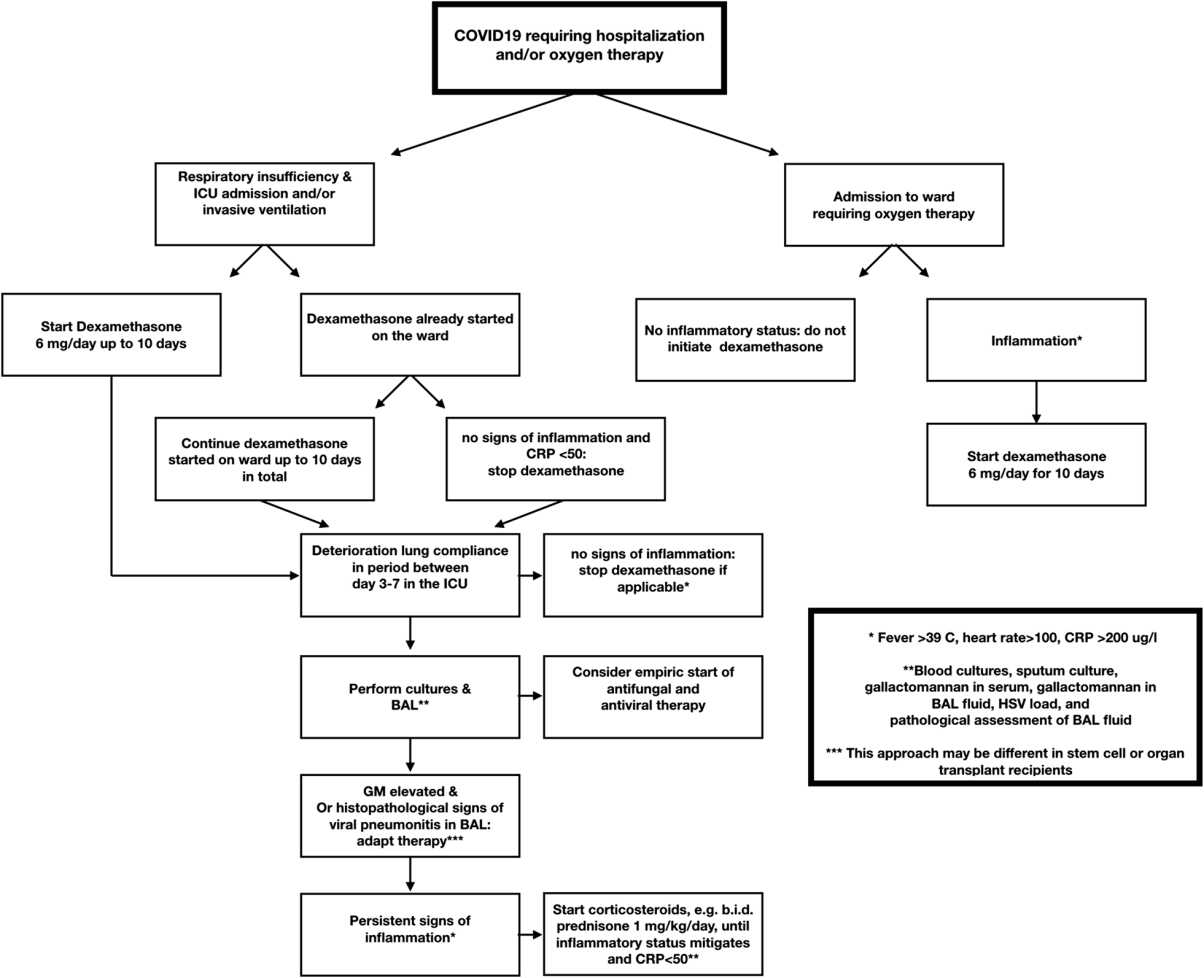
Flowchart with suggested diagnostic approach of secondary infections and the use of steroids in COVID-19-associated ARDS (15)

In the anti-inflammatory subphenotype, there is probably not only a lack of benefit, but also a potential for harm if corticosteroids are applied in the absence of inflammation. This is illustrated by a recent retrospective analysis on the effects of dexamethasone in COVID-19 [39]. If patients had a C-reactive protein (CRP) ≥ 200 mg/l, there was a high potential for benefit of corticosteroids in COVID-19 ARDS with a lower mortality (OR 0.23; 95% CI 0.08–0.70). In contrast, if CRP levels were only mildly elevated (≤ 100), this benefit dissipated and mortality increased (OR 2.64; 95% CI 1.39–5.03). Furthering this hypothesis, one may hypothesize (Fig. 1) that dexamethasone indeed mitigates the proinflammatory response at alveolar level in a subgroup of COVID-19, but it may also tip the balance toward anti-inflammatory aspects in a subset of patients (Fig. 2). Those patients may be more prone to outgrowth of Aspergillus spp. or herpes simplex reactivations (Fig. 3.) [40–42]. We propose a more practical approach to tailored therapy in COVID-19-associated ARDS (Fig. 4). The premise is that timely treatment with corticosteroids is essential in the treatment of COVID-19 but that it has no added value if systemic inflammation is lacking. Ideally, an extensive analysis of alveolar cytokine levels inflammatory response and degree of fibrosis could guide tailored therapy. Analysis of this alveolar inflammatory response has been performed in small patient groups, showing a distinct difference with systemic inflammatory response [43, 44]. However, such analysis is hardly available in usual clinical practice. In addition, it is unclear how these results should be interpreted when initiating immunomodulatory therapy. This means that, in principle, corticosteroids are started or continued if they had already been started in COVID-19 patients on the ward. If there are no signs of inflammation (i.e., CRP < 50, low-dose vasopressor and the absence of fever, tachycardia and tachypnea) immediately upon admission to the ICU, the corticosteroids may probably be discontinued. In addition, we argue for extra vigilance of secondary infections as a result of the use of immune-modulating therapy, especially in respiratory deterioration after 3–7 days after initiation of corticosteroids. In such a case, bronchoalveolar lavage (BAL) should be performed with specific attention for signs and symptoms of infections caused by opportunistic pathogens. In case of systemic inflammation (e.g., CRP ≥ 200, tachycardia, high-dose vasopressor, fever, tachycardia and tachypnea) and deteriorating lung compliance despite the absence of positive fluid balances, corticosteroids should be continued or even increased in case of aggravating signs of inflammatory response while awaiting results of extensive cultures including bronchoalveolar lavage (BAL) [15]. Empirically starting antifungals agents and/or antivirals agents may be considered in this setting as well, depending on the clinical status of the patient.

In addition, one may also hypothesize a rationale for IFN beta-1b treatment in the patients with an anti-inflammatory phenotype. Results of treatment with IFN beta-1b in COVID-19 patients have been conflicting, i.e., benefit in combination with anti-viral drugs in one small study and no mortality reduction in the SOLIDARITY trial [45–47]. Despite the apparently lower levels of type II interferons in severe COVID-19, IFNy has not been applied in a clinical trial up to now [48]. The lack of beneficial effect of IFN beta-1b in combination with glucocorticoids in a large non-selected COVID-19 population might be explained by failure to stratify for inflammatory status. Studies should be designed and executed to evaluate the potential role of type I and type II interferons in the setting of dexamethasone-treated COVID-19 patients.

## Conclusion

The use of corticosteroids in COVID-19 patients has increased significantly since the publication of the RECOVERY trial [11]. However, the generalized non-tailored use of corticosteroids might tip the balance to a subset of patients with anti-inflammatory aspects, necessitating a more tailored therapy with vigilance for reactivation of HSV and/or secondary infections with Aspergillus spp.

## Data Availability

Not applicable.
